# Immunotherapy in endometrial cancer - an evolving therapeutic paradigm

**DOI:** 10.1186/s40661-015-0020-3

**Published:** 2015-12-02

**Authors:** Teresa C. Longoria, Ramez N. Eskander

**Affiliations:** University of California, Irvine Medical Center, 101 The City Drive South, Bldg 56, Ste 800, Orange, CA 92868 USA

**Keywords:** Adoptive cellular therapy, Bispecific T-cell engager antibodies, Endometrial cancer, Immune checkpoint inhibitors, Therapeutic vaccination, Tumor microenvironment

## Abstract

Endometrial cancer is the only gynecologic malignancy with a rising incidence and mortality. While cure is routinely achieved with surgery alone or in combination with adjuvant pelvic radiotherapy when disease is confined to the uterus, patients with metastatic or recurrent disease exhibit limited response rates to cytotoxic chemotherapy, targeted agents, or hormonal therapy. Given the unmet clinical need in this patient population, exploration of novel therapeutic approaches is warranted, and attention is turning to immunomodulation of the tumor microenvironment. Existing evidence suggests that endometrial cancer is sufficiently immunogenic to be a reasonable candidate for active and/or passive immunotherapy. In this review, we critically examine what is known about the microenvironment in endometrial cancer and what has been learned from preliminary immunotherapy trials that enrolled endometrial cancer patients, encouraging further attempts at immunomodulation in the treatment of aggressive forms of this disease.

## Background

As the most common cancer of the female genital tract, it is estimated that in 2015 endometrial cancer will be diagnosed in over 54,000 women and will be responsible for over 10,000 deaths in the United States [1]. At the time of diagnosis, 67 % of women have disease confined to the uterus and an associated 5-year survival rate of 95 % [1]. In contrast, the 8 % of patients with distant metastases at the time of diagnosis have a 5-year survival rate of 17 % [1] and face the prospect of cytotoxic chemotherapy (primarily with taxanes, anthracyclines, and platinum drugs) with limited response.

Since the completion of Gynecologic Oncology Group (GOG) protocol 177, which explored the triplet regimen of paclitaxel, doxorubicin and cisplatin (TAP) in patients with advanced stage and recurrent endometrial cancer, demonstrating an overall response rate of 57 % and median overall survival of 15.3 months, results have been clinically disappointing. Furthermore, the toxicity associated with the 3-drug regimen has limited its clinical utility [2]. The GOG, in the 229 queue, has evaluated a series of targeted agents [3–9] including bevacizumab (229E), aflibercept (229 F), bevacizumab/temsirolimus (229G), AZD6244 (229H), brivanib (229I), cediranib (229 J), AMG386 (229 L) and BIBF 1120 (229 K) with modest overall response rates, ranging from 0 % - 24.5 % (Table 1). Hormonal therapy is better tolerated but results in response rates between 18 % and 34 % [10]. With taxanes alone showing response rates of greater than 20 % in select patients (taxane-naïve) with recurrent disease [11, 12], effective second-line chemotherapeutic options are limited. Given the above unmet clinical need, exploration of novel therapeutic approaches is warranted in this patient population.

**Table 1:** Clinical end points in the GOG 229 queue

GOG Trial	N	ORR	PFS > 6mo	Median PFS (mo.)	Median OS (mo.)
229 N [7]	28	0 %	11 %	2.1	9.4
229 K [6]	37	9.4 %	22 %	3.3	10.1
229 I [8]	45	18.6 %	30 %	3.3	10.7
229 G* [5]	53	24.5 %	47 %	5.6	16.9
229 F* [4]	49	8.9 %	40 %	2.9	14.6
229 E [3]	56	13.5 %	40.4 %	4.2	10.5
229 J [9]	53	12.5 %	29 %	3.5	12.5

*Significant Grade 3/4 adverse events were encountered on these studies preventing subsequent development of a phase 3 trial; GOG = gynecologic oncology group; ORR = overall response rate; PFS = progression free survival; OS = overall survival

Within cancer drug development, a shift in focus from the tumor cell itself to the tumor microenvironment (TME) has been gradually gaining momentum. This shift has come with the recognition of the limitations of targeted therapy, which act by blocking essential biochemical pathways or mutant proteins that are required for tumor cell growth and survival. The ideal use of targeted therapies is in cancers with a single dominant driver mutation and a small mutational load, the classic example being chronic myeloid leukemia (CML) bearing the Philadelphia chromosome (bcr-abl gene translation) [13]. Most cancers, however, exhibit genetic heterogeneity. Fortunately, this same genetic heterogeneity that translates into limited therapeutic responses with targeted agents, may result in enhanced tumor immunogenicity, provoking an adaptive immune response. This concept of tumor immunogenicity is well appreciated for its role in determining the efficacy of immunotherapy [14]. Currently, our understanding of the somatic mutational load in endometrial cancer is evolving, and work is being done to identify the correlation between mutations and immunogenicity [15].

In this review, we critically examine what is known about the microenvironment in endometrial cancer and what has been learned from preliminary immunotherapy trials that enrolled endometrial cancer patients, encouraging further attempts at immunomodulation in the treatment of aggressive forms of this disease.

### Characterizing the Tumor Microenvironment

Exploiting the immune system in cancer therapeutics relies on characterizing its components within the TME. This has proven to be a major endeavor, given the differences in the immune cell composition between different types of cancer, as well as between cancers of the same type. This diversity results from the phenotypic and functional plasticity of immune cells, which are responsible for a diverse set of tasks within the immune system’s overarching objective of host protection and tissue homeostasis. In both innate and adaptive immunity, immune cells are simultaneously responsible for promoting host defense while limiting collateral tissue damage. It is well established that the microenvironment has the capacity to regulate the phenotype and function of differentiated myeloid or lymphoid cells at the level of their progenitors, during their lineage-specific differentiation, and after they have matured into the fully differentiated cell types [16]. This plasticity lends support to the idea that differentiated hematopoietic cells should be viewed on a dynamic continuum rather than in distinct subcategories.

The lack of success in developing a cohesive picture of endometrial cancer on the molecular level may be explained, in part, by the fluctuations in immune cell composition of the endometrium that result from hormonal influences. As described in two recent reviews [17, 18], the immune system within the endometrium faces a unique challenge; it must be competent enough to provide protection against sexually transmitted pathogens while being permissive enough to allow the development of an allogeneic fetus. As such, this site within the female reproductive tract has evolved in such a way that sex hormones precisely regulate immune function to accomplish both tasks. The number of macrophages, neutrophils, and natural killer (NK) cells steadily increase throughout the menstrual cycle and are most abundant before menstruation, perhaps reflecting their role in the breakdown of the endometrium and in host defense during disruption of the mucosal barrier. Similarly, adaptive immune cells, which are present in the endometrium as unique aggregates consisting of a B-cell core surrounded by T cells and an outer halo of macrophages, increase in number throughout the proliferative phase and temporarily lose cytotoxic capabilities during the secretory phase, when conception may occur. These findings highlight the exceptional responsiveness of the immune system to hormonal fluctuations in this particular microenvironment.

In regards to the composition of the TME and corresponding associations with prognosis, endometrial cancer has been relatively understudied in comparison to other malignancies. In ovarian cancer, for example, it is well established that the presence of intraepithelial tumor infiltrating lymphocytes (TILs) is a robust predictor of a more favorable outcome, as demonstrated in a recent meta-analysis including 10 studies [19]. Early clinicopathologic studies were conflicting in regards to whether TILs were more common in low-grade [20] vs. high-grade endometrial cancers [21] and whether the location of TILs has prognostic implications. A perivascular lymphocytic infiltrate has been shown to correlate with poor overall survival (OS) on univariate analysis [22] while an intraepithelial lymphocytic infiltrate at the invasive border [23] has been shown to correlate with improved OS on multivariate analysis. On the whole, given the limited data set, and study heterogeneity, comparing studies to draw meaningful conclusions has been problematic.

Contemporary studies have not only examined the presence of intra-tumoral, cytotoxic T cells but also the ratio of CD8+ TILs to regulatory T cells (Tregs: CD4+ CD25+ FOXP3+), which are well known for their physiologic role in peripheral tolerance and their pathological role in antitumor immunity. After adjusting for well known prognostic factors in multivariate analysis, de Jong and colleagues [24] found that the presence of high numbers of CD8+ TILs was an independent predictor of increased OS (whole cohort and type II) and that the presence of a high CD8+/FoxP3+ ratio was an independent predictor of increased disease-free survival (DFS) in type I, though not type II, endometrial cancer patients. The importance of the ratio of CD8+ to FoxP3+ T cells to DFS was confirmed in an additional study that did not stratify by tumor type [25]. The amount of intra-tumoral Tregs alone has not been shown to impact recurrence and survival curves [26, 27], though a statistically significant correlation has been shown between the presence of Tregs and tumor stage, grade, and presence of myometrial invasion [28].

There appears to be a better understanding of the role of myeloid cells, in comparison to lymphoid cells, in endometrial pathology. While myeloid-derived suppressor cells (MDSCs) have been detected in tumor specimens [29], tumor associated macrophages (TAMs) have been consistently identified as the dominant contributor to a pro-tumorigenic environment [30]. TAM density, particularly within the stromal compartment, has been shown to steadily increase with disease progression from precancerous endometrial lesions (various forms of hyperplasia) to endometrial cancer [31, 32]. The presence of TAMs has been repeatedly correlated to aggressive features within the primary tumor, specifically higher stage and grade and the presence of lymphovascular and myometrial invasion [27, 33–36]. The results of only one study, containing a small and heterogeneous cohort of type I and II carcinomas, has differed from these consistent findings [31]. Additionally, the presence of TAMs has been strongly associated with pelvic lymph node metastases [27, 32, 35, 37] and an angiogenic profile [34–36, 38, 39].

Recently, Kubler and colleagues [27] were the first to demonstrate that TAM density is an independent prognostic factor for recurrence-free survival, finding that a high density compared to a low density of TAMs increased the risk of recurrence by a factor of 8.3. In their study, a significant relationship between the presence of TAMs and overall survival was found on univariate analysis but not on multivariate analysis. They hypothesize that detecting significance may not have been possible in this cohort of patients of mostly early stage disease due to the length of follow-up and the treatment of relapsed cases with curative intent. These same factors may explain why previous studies were only able to document a trend towards significance [32, 33, 35].

### Immunotherapy in Endometrial Cancer

#### Therapeutic Vaccination

Therapeutic cancer vaccination is a form of active immunotherapy (Table 2). Active immunotherapies stimulate the host’s own immune system to mount an anti-tumor immune response and induce immunological memory, theoretically producing a durable effect after treatment is stopped. Cancer vaccines exploit the cellular arm of the immune system, inciting a cytotoxic T-lymphocyte response against tumor-associated antigens (TAAs). In concept, cancer vaccines offer the prospect of high specificity, low toxicity, and prolonged activity, though these properties have yet to be reliably translated in clinical practice [40].

**Table 2:**
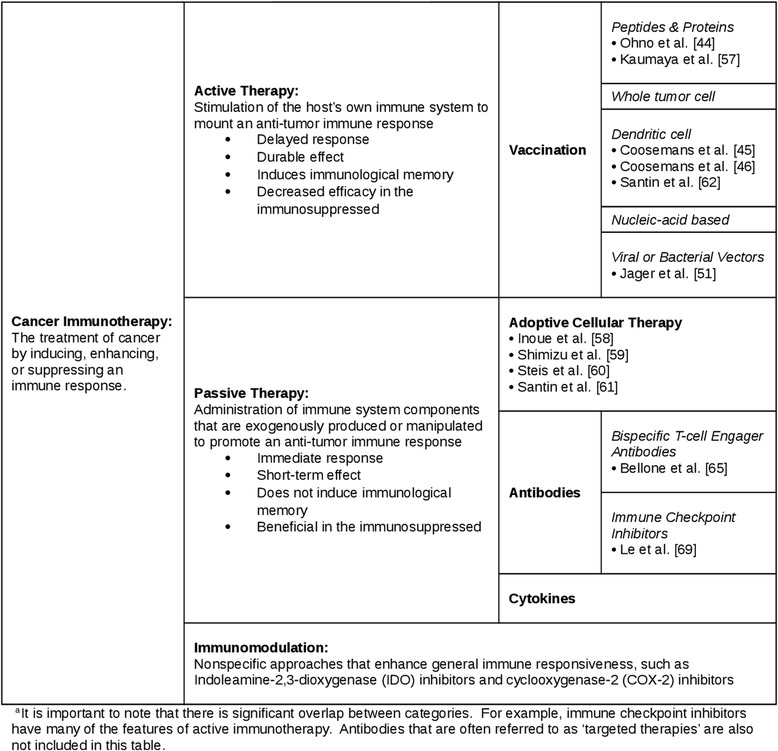
Immunotherapeutic approaches and their application to endometrial cancer^a^

The product of Wilms tumor gene 1 (WT1) has been identified as a TAA with therapeutic potential in endometrial cancer. WT1 is located on chromosome 11p13 and encodes a transcription factor that plays an essential role in the normal development of the urogenital system. It has been detected in 0 % to 79 % of endometrial cancer, depending on the immunohistochemical staining technique [41]. The safety and tolerability of a weekly WT1 peptide vaccine (HLA-A2402-restricted, modified 9-mer WT1 peptide emulsified with Montanide ISA51 adjuvant) in 12 patients with recurrent or progressive gynecologic malignancies was demonstrated in a recent phase I trial [42]. Adverse events were limited to erythema at the injection site, and the disease control rate in the initial 3 months was 25 % (stable disease [SD] in 3 patients, progressive disease [PD] in 9 patients). Unfortunately, the only subject with recurrent uterine carcinosarcoma failed to respond to treatment, with disease progression after 11 vaccine injections (3 months on therapy). Utilizing an alternative approach, Cooseman and colleagues have reported the vaccination of 4 patients with advanced serous endometrial cancer with autologous dendritic cells loaded with WT1 mRNA [43, 44]. After 4 weekly injections, 3 of 4 patients demonstrated an immunological response (defined as an increase in the percentage of WT1-specific T-cells or NK cells among peripheral blood mononuclear cells), 2 of 4 patients demonstrated a molecular response (defined as a decrease in CA-125), but no patients were found to have a decrease in tumor size on repeat CT scan. Similar to the experience in the WT1 peptide trial, adverse events were limited to erythema at the injection site and 1 local allergic reaction. The investigators hypothesized that the limited clinical response may be partially attributable to the advanced stage of the patients and the early termination of therapy once radiological progression was demonstrated.

Cancer testis (CT) antigens, expressed exclusively in male germ cells and placental tissue in healthy adults but ectopically in tumor cells of multiple types of human cancer, have emerged as excellent candidates for therapeutic manipulation. The restricted nature of their expression lends to high tumor-specificity and immunogenicity [45]. To date, several CT antigens have been identified in endometrial cancer. NY-ESO-1 and MAGE-A4 have been reported in 19 % and 12 % of endometrioid adenocarcinomas, respectively [46]. These numbers increase to 32 % and 63 % of USC, respectively. Additionally, KU-CT-1 has been identified in 64 % of cases of endometrial cancer [47] and SSX-4 in 24 % of cases [48]. In a two-part, open-label cohort study designed to test the safety and immunogenicity of recombinant vaccinia-NY-ESO-1 and recombinant fowlpox-NY-ESO-1, 36 patients with a wide range of tumor types experienced a similar, minor reaction to the vaccine (erythema and pruritis at the injection site) but differed significantly in their immunologic response [49]. The sole patient with endometrial cancer was one of three patients to demonstrate NY-ESO-1 sero-conversion and both a CD8+ and CD4+ T-cell response.

Additionally, human epidermal growth factor receptor 2 (HER-2/neu), the transmembrane receptor encoded by the *ERBB2* gene, has exciting potential in the treatment of endometrial cancer. In USC, specifically, overexpression of HER-2/neu ranges from 16 % to 80 % and is associated with worse overall survival [50–52]. The success of targeted therapy with trastuzumab, a recombinant humanized monoclonal antibody against HER2, in producing impressive response rates and prolonged disease-free survival in patients with metastatic breast cancer [53, 54] has encouraged the development of active immunotherapies that may produce a more durable anti-tumor immune response. In a phase I, dose-escalating, safety trial in patients with various metastatic, heavily pretreated cancers, Kaumaya and colleagues [55] tested a novel peptide combination vaccine consisting of 2 B-cell epitopes derived from the HER2 extracellular domain. In utilizing B-cell epitopes rather than T-cell epitopes, they were able to overcome the requirement for specific HLA restrictions in their patient population and engage the humoral arm of the immune system. Between the 2 endometrial cancer patients enrolled in the study, 1 patient has a partial response, experiencing extended clinical benefit at 4 years after the initial vaccination. In functional studies, the vaccine elicited antibodies in this patient that disrupted 2 different HER2 signaling methods, ultimately suppressing HER2 phosphorylation and inhibiting cell proliferation. Vaccines such as this one offer hope that we may overcome the limitations of antibody therapy, namely the short half-life of IgG, requiring frequent treatments and accruing high costs.

#### Adoptive Cellular Therapy

Adoptive cellular therapy is a form of passive immunotherapy (Table 2). Passive immunotherapies involve the administration of immune system components (i.e. antibodies, cytokines, lymphocytes) that are exogenously produced or manipulated to promote an anti-tumor immune response. Unable to induce immunological memory, they offer immediate but short-term protection. In adoptive cellular therapy, cells from the blood or bone marrow are isolated, activated and expanded in vitro, and re-infused into the same patient (autologous) or a different patient (allogeneic). The technology has evolved substantially and now includes the generation of tumor-reactive T cells that are genetically engineered to express recombinant or chimeric T-cell receptors directed against common TAAs (CAR T cells).

Adoptive cellular therapy in the treatment of endometrial cancer has not yet exploited these most recent technological advances. The earliest animal studies involved the infusion of lymphokine-activated killer (LAK) cells with and without additional immuno-stimulatory components (Il-2, lentinan) [56, 57]. This therapy produced growth retardation of tumor in nude mice. Intraperitoneal adoptive transfer of LAK cells with IL-2 has also been tested in a phase I trial that enrolled 12 colorectal cancer patients, 10 ovarian cancer patients, and 1 endometrial cancer patient with abdominal metastases [58]. Thirty percent of patients had a laparoscopy- or laparotomy-documented PR, though this did not include the patient with endometrial cancer. While the majority of adverse events (minor to moderate hypotension, fever, chills, rash, nausea, vomiting, abdominal pain and distension, diarrhea, oliguria, fluid retention, thrombocytopenia, and minor elevations of liver function tests) were attributable to Il-2, intraperitoneal fibrosis (14 patients) was a notable toxic side effect of the therapy that led to treatment discontinuation in 5 patients. Adding to the question of safety, one patient had a grand mal seizure and another had colonic perforation.

The infusion of peripheral blood T cells stimulated with tumor lysate-pulsed autologous dendritic cells has been reported by Santin and colleagues [59] in a 65-year-old patient with advanced, chemoresistant endometrial cancer. Prior to the treatment, which consisted of 3 infusions administered every 3 to 4 weeks, the patient’s liver metastasis had substantially increased in size (9.5 X 8 cm to 14 X 10 cm in 3 weeks). During treatment, stabilization of the liver metastasis was achieved as a result of a tumor-specific, cytotoxic T-cell response. A more dramatic response was likely limited by the inability of the activated T cells to deeply infiltrate the large tumor mass, as evaluated in 3 dimensions by single photon emission computerized tomography (SPECT) imaging. Since publication of this report, these investigators have also demonstrated the ability to induce a tumor-specific, cytotoxic T-cell response in vitro through vaccination with tumor lysate-pulsed autologous dendritic cells in 3 patients with USC [60].

#### Bispecific T-cell Engager (BiTE) Antibodies

The diverse array of molecules employed within passive immunotherapeutic approaches now includes bispecific T-cell engager (BiTE) antibodies [61]. These novel molecules induce a transient cytolytic synapse between a cytotoxic T cell and the cancer target cell. This interaction results in discharge of cytotoxic T-cell contents following perforin fusion with the T-cell membrane resulting in direct tumor cell lysis. Currently, the only drug within this class with United States FDA approval is blinatumomab (BiTE for CD 19 and CD3) for patients with acute lymphoblastic leukemia (ALL), based on an impressive complete remission rate in a phase 2 clinical trial [62].

Solitomab, which targets epithelial-cell-adhesion-molecule (EpCAM) on tumor cells while also containing a CD3 binding region, is being pursued as treatment for metastatic, recurrent, or persistent USC overexpressing EpCAM (86 % of USC cell lines tested by flow cytometry) [63]. After exposure to peripheral blood lymphocytes in vitro, EpCAM positive USC cells were found to be resistant to NK or T-cell-mediated killing. This resistance was overcome by incubating the cell lines with solitomab. Additionally, ex vivo incubation of autologous tumor associated lymphocytes (TAL) with EpCAM expressing malignant cells in ascites with solitomab resulted in a significant increase in both CD4+ and CD8+ T-cell proliferation, an increase in T-cell activation markers, and a reduction in number of viable USC cells in ascites.

#### Immune Checkpoint Inhibitors

The therapies discussed thus far involve activating the immune system to achieve tumor cell death. However, recognition that the effectiveness of both active and passive immunotherapies are reduced by tumor immune evasion [40] has led to a recent paradigm shift within immunotherapeutics away from a focus on stimulating the immune system to a focus on inhibiting the inhibitors of an adequate immune response. Among the emerging strategies of tackling immune tolerance, immune checkpoint inhibitors are the most promising.

Immune checkpoints refer to a variety of inhibitory pathways employed by the immune system to maintain self-tolerance and minimize collateral damage during physiologic responses to pathogens. Many of these pathways are initiated by ligand-receptor interactions on the surface of immune cells and, thus, are logical targets for monoclonal antibodies. Cytotoxic T-lymphocyte-associated protein-4 (CTLA-4) and programmed cell death protein-1 (PD-1) were the first, and remain the most relevant, immune-checkpoint receptors to be clinically targeted [64]. Although PD-1 and CTLA-4 belong to the same CD28 family of T-cell receptors, they assume very different roles in the down regulation of an inflammatory response. While CTLA-4 predominately regulates T cell activation within secondary lymphoid organs, PD-1 predominately regulates T cell effector function within peripheral tissues.

Importantly, immunohistochemical studies on endometrial cancer specimens have detailed PD-1 and PD-L1 expression levels surpassing those seen in ovarian and cervical carcinoma. Specifically, Vanderstraeten et al. described PD-L1 expression levels of 67-100 % in primary, recurrent and metastatic endometrial cancer specimens [29]. At the 2015 annual meeting of the Society of Gynecologic Oncology, Herzog et al. reported PD-1 expression levels of 75 %, and PD-L1 expression levels ranging from 25-47 %, once again surpassing all examined cervical and ovarian cancer specimens [65] (Table 3). Given the above, investigation of immune checkpoint inhibitors in patients with metastatic and recurrent endometrial cancer may represent a promising alternative to traditional cytotoxic therapies.

**Table 3:** PD-1 and PD-L1 expression levels in uterine cancer (450 specimens) [67]

Histology	PD-1	PD-L1
	% Expression based on IHC staining*	
Endometrioid	77.9	39.7
Serous Carcinoma	68.2	10.2
Carcinosarcoma	80.0	22.2
Leiomyosarcoma	46.9	36.0
Stromal Sarcoma	64.3	64.3
Clear Cell Carcinoma	69.2	23.1

* IHC antibody = Spring Bioscience (Rabbit anti-Human IgG)

While preliminary evidence exists that tumor cell surface PD-L1 expression correlates with the likelihood of response to PD-1 pathway inhibition [66], the best argument for the use of checkpoint inhibitors in select endometrial cancer cases was recently put forth by a phase 2 trial of pembrolizumab, a humanized monoclonal antibody to the PD-1 receptor, in patients with mismatch repair- (MMR-) deficient tumors [67]. This trial was designed to test the hypothesis that MMR-deficient tumors are more responsive to PD-1 blockade than MMR-proficient tumors, due to the high somatic mutational load, resulting in neoantigen formation and a more prominent lymphocytic infiltrate. As predicted, the two cohorts with MMR-deficient cancers (one with colorectal cancer patients and the other with non-colorectal cancer patients, including 2 patients with endometrial cancer) had significantly higher objective response rates by immune-related response criteria and by Response Evaluation Criteria in Solid Tumors (RECIST). They also had a significantly better immune-related PFS at 20 weeks and disease control rate by RECIST. Interestingly, patients with sporadic MMR-deficient tumors responded more frequently to treatment than those with Lynch syndrome (100 % vs 27 %). This study provides preliminary clinical evidence that immune checkpoint inhibitors may be used effectively in the treatment of MMR-deficient endometrial cancers, and trials exploring this hypothesis are currently in development.

More recently, Howitt et al. specifically examined the hypothesis that microsatellite unstable endometrial cancers would exhibit more tumor specific neoantigens, resulting in increased tumor infiltrating lymphocytes and a compensatory up-regulation of immune checkpoints (9). Microsatellite unstable tumors exhibited higher numbers of CD3+ and CD8+ tumor infiltrating lymphocytes. Furthermore, PD-1 was overexpressed in tumor infiltrating lymphocytes, and peri-tumoral lymphocytes of microsatellite unstable tumors.

## Conclusion

Despite existing evidence that endometrial cancer, particularly the most aggressive forms of the disease, is sufficiently immunogenic to be a reasonable candidate for immunomodulation, attempts to expand the role of active and/or passive immunotherapy in the treatment of this condition have been limited. At a time when the U.S. FDA-approved indications for immune checkpoint inhibitors is steadily amassing, progress in the endometrial cancer arena has been slow. Uniquely, endometrial cancer is the only gynecologic cancer with a rising incidence and mortality, and identifying effective therapies for patient with metastatic or recurrent disease is critical.

As reviewed here, these patients have been enrolled in small preclinical and phase I trials assessing the utility of immunotherapy. These studies have demonstrated encouraging immunologic responses but few clinical responses, provoking questions regarding the ability to establish therapeutic efficacy in a small number of heavily pretreated patients with advanced disease and short follow-up. The most urgent questions in identifying the utility of immunotherapy for the treatment of endometrial cancer are: how do we identify the subset of individuals most likely to respond to immunotherapy, the biomarkers most likely to predict successful treatment, and the therapy combinations most likely to enhance drug performance while limiting toxicity.

## Abbreviations

ALL: Acute lymphoblastic leukemia
BiTE antibody: Bispecific T-cell engager antibody
CML: Chronic myeloid leukemia
CR: Complete responses
CT antigen: Cancer testis antigen
CTLA-4: Cytotoxic T-lymphocyte-associated protein-4
DFS: Disease-free survival
EpCAM: Epithelial-cell-adhesion-molecule
GOG: Gynecologic Oncology Group
HER-2/neu: Human epidermal growth factor receptor 2
LAK cells: Lymphokine-activated killer cells
MDSCs: Myeloid-derived suppressor cells
MMR: Mismatch repair
NK cells: Natural killer cells
OS: Overall survival
PD: Progressive disease
PD-1: Programmed cell death protein-1
RECIST: Response Evaluation Criteria in Solid Tumors
PR: Partial responses
SD: Stable disease
SPECT: Single photon emission computerized tomography
TAAs: Tumor-associated antigens
TALs: Tumor associated lymphocytes
TAMs: Tumor associated macrophages
TAP: Paclitaxel, doxorubicin and cisplatin
TILs: Tumor infiltrating lymphocytes
TME: Tumor microenvironment
Tregs: Regulatory T cells
WT1: Wilms tumor gene 1
