# Erratum to: Immunotherapy in endometrial cancer - an evolving therapeutic paradigm

**DOI:** 10.1186/s40661-016-0021-x

**Published:** 2016-02-17

**Authors:** Teresa C. Longoria, Ramez N. Eskander

**Affiliations:** University of California, Irvine Medical Center, 101 The City Drive South, Bldg 56, Ste 800, Orange, CA 92868 USA

Unfortunately, after publication of the article [1] it was noticed that Table 2 (Table 1 here) was formatted incorrectly during the production process. The corrected table can be seen below and the original article has been updated to reflect this change.

**Table 1:**
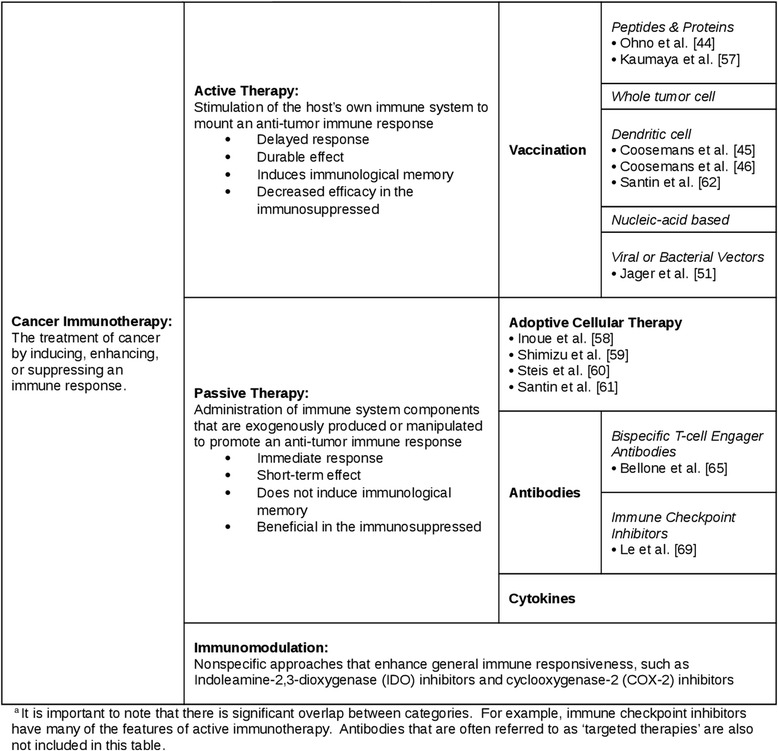
Immunotherapeutic approaches and their application to endometrial cancer^a^
